# Supplementary material for: Emoticon-Based Ambivalent Expression: A Hidden Indicator for Unusual Behaviors in Weibo
Source: PLoS One. 2016 Jan 22;11(1):e0147079. doi: 10.1371/journal.pone.0147079 (PMC4723056; doi:10.1371/journal.pone.0147079)
Supplement: S1 Datasets — All the datasets collected from Weibo can be freely downloaded from the permanent location in figshare.com: https://dx.doi.org/10.6084/m9.figshare.2060046. (PDF) [file pone.0147079.s001.pdf]

PLOS ONE Supporting Information

Supporting Information Captions

S1 Datasets. The datasets download location. All the datasets collected from Weibo can be freely downloaded from the permanent location in figshare.com: https://dx.doi.org/10.6084/m9.figshare.2060046.
