# Supplementary material for: Emoticon-Based Ambivalent Expression: A Hidden Indicator for Unusual Behaviors in Weibo
Source: PLoS One. 2016 Jan 22;11(1):e0147079. doi: 10.1371/journal.pone.0147079 (PMC4723056; doi:10.1371/journal.pone.0147079)
Supplement: S1 Table — (PDF) [file pone.0147079.s002.pdf]

PLOS ONE Supporting Information

Supporting Information Captions

S1 Table. The entire list of emoticons and their descriptions.

**Positive**			**Negative**		
**Emoticon**	**Chinese Description**	**Description**	**Emoticon**	**Chinese Description**	**Description**
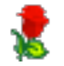	鲜花	Fresh flower	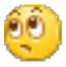	白眼	Rolling his eye
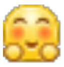	太开心	Too happy	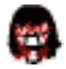	咆哮	Roar
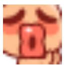	亲一个	Kiss	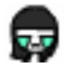	哭泣女	Crying girl
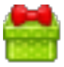	礼物	Gift	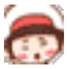	悲剧	Tragedy
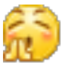	鼓掌	Applaud	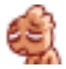	悲催	Misery
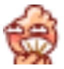	偷乐	Steal happiness	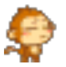	叹气	Sigh
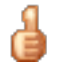	赞	Like	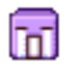	哭	Cry
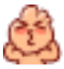	给劲	Cool	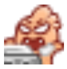	躁狂症	Irritable
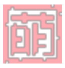	萌	Cute	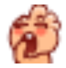	困死了	Tired
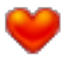	心	Heart	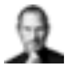	悼念乔布斯	Mourning Jobs
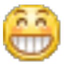	嘻嘻	Smirk	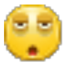	困	Tired
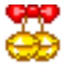	圣诞铃铛	Christmas bell	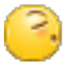	右哼哼	Despise
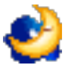	月亮	Moon	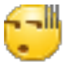	黑线	Awkward

Positive			Negative		
Emoticon	Chinese Description	Description	Emoticon	Chinese Description	Description
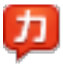	给力	Awesome	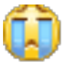	泪	Tears
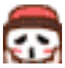	做鬼脸	Make a face	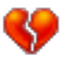	伤心	Heart broken
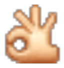	OK	OK	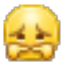	委屈	Aggrieved
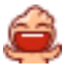	笑哈哈	Laughingly	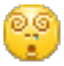	晕	Dizzy
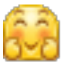	抱抱	Hug	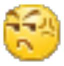	哼	Snort
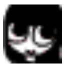	吐舌头	Sticking tongue out	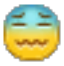	生病	Sick
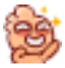	噢耶	Ooh, yeah!	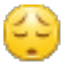	失望	Disappointed
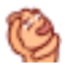	转发	Repost	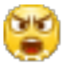	怒骂	Rant
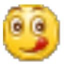	馋嘴	Hungry	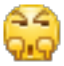	鄙视	Look down upon
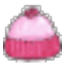	温暖帽子	Warm hat	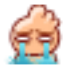	泪流满面	Cry
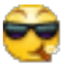	酷	Cool	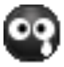	衰	Bad luck
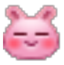	兔子	Rabbit	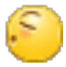	左哼哼	Despise
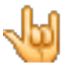	Haha	Complacent	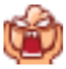	崩溃	Collapse
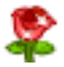	玫瑰	Rose	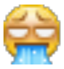	吐	Throw up

PLOS ONE Supporting Information

Positive			Negative		
Emoticon	Chinese Description	Description	Emoticon	Chinese Description	Description
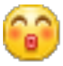	亲亲	Kiss	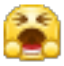	抓狂	Freak out
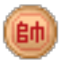	帅	Handsome	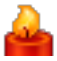	蜡烛	Candle
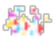	礼花	Fireworks	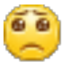	悲伤	Sad
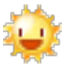	太阳	The Sun	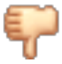	弱	Lousy
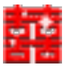	喜	Happiness	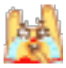	大哭	Cry
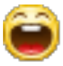	哈哈	Ha-ha	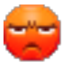	怒	Angry
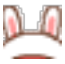	冒个泡	Say hi	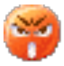	愤怒	Rage
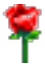	花	Flower	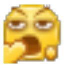	打哈气	Boring
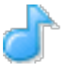	音乐	Music			
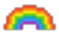	彩虹	Rainbow			
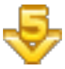	威武	Mighty			
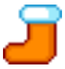	圣诞袜	Christmas socks			
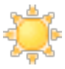	阳光	Sunshine			
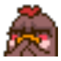	爱你哦	Love you			

PLOS ONE Supporting Information

Positive			Negative		
Emoticon	Chinese Description	Description	Emoticon	Chinese Description	Description
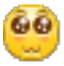	爱你	Love you			
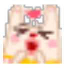	开心	Happy			
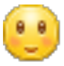	可爱	Lovely			
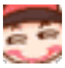	猥琐	Mock			
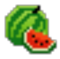	西瓜	Watermelon			
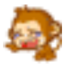	哇哈哈	Funny			
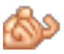	来	Come on			
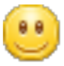	微笑	Smile			
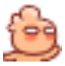	带感	Great			
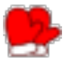	手套	Glove			
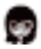	春暖花开	Spring blossoms			
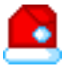	圣诞帽	Christmas hat			
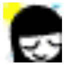	乐乐	Happy			
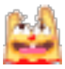	大笑	Laugh			

PLOS ONE Supporting Information

Positive			Negative		
**Emoticon**	**Chinese Description**	**Description**	**Emoticon**	**Chinese Description**	**Description**
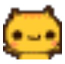	Din 推撞	Play			
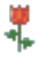	跳舞花	Dancing flower			
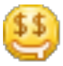	钱	Money			
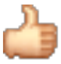	Good	Good			
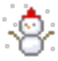	雪人	Snowman			
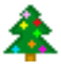	圣诞树	Christmas tree			
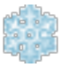	雪	Snowflake			
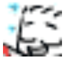	欢欢	Happy			
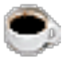	咖啡	Coffee			
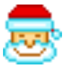	圣诞老人	Santa Clause			
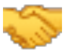	握手	Handshake			
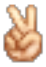	耶	Yeah			
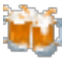	干杯	Cheers			
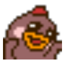	好得意	Complacent			

Positive			Negative		
Emoticon	Chinese Description	Description	Emoticon	Chinese Description	Description
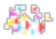	礼花	Fireworks			
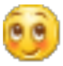	害羞	Shy			
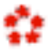	爱	Love			
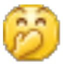	偷笑	Titter			
No longer Avaiable	呵呵	Funny			
No longer Avaiable	加油	Fighting			
No longer Avaiable	花心	Fond of			
