# Supplementary material for: Emoticon-Based Ambivalent Expression: A Hidden Indicator for Unusual Behaviors in Weibo
Source: PLoS One. 2016 Jan 22;11(1):e0147079. doi: 10.1371/journal.pone.0147079 (PMC4723056; doi:10.1371/journal.pone.0147079)
Supplement: S2 Table — (PDF) [file pone.0147079.s003.pdf]

PLOS ONE Supporting Information

Supporting Information Captions

S2 Table. Key terms for positive events.

A: Jan 1, 2012. New Year

New Year(新年), happy(快乐), one year(一年), the first day(第一天), hope(希望), ha-ha(哈哈), 2011, happiness(幸福), together(一起) , joyful(开心), cross year(跨年)

B: Jan 22, 2012. Eve of lunar New Year

New year(新年), happy(快乐), pay New Year's call(拜年), Spring Festival Gala(春晚), Year of Dragon(龙年), one year(一年), happiness(幸福), celebrate the spring festival(过年), healthy(健康), friends(朋友)

C: Jan 23, 2012. Spring Festival

New year(新年), happy(快乐), pay New Year's call(拜年), Year of Dragon(龙年), ha-ha(哈哈), healthy(健康), one year(一年), happiness(幸福), first(第一), this year(今年)

D: Feb 6, 2012. Lantern Festival

happy(快乐), Lantern Festival(元宵节), rice dumpling(汤圆), happiness(幸福), firework(烟花), Chinese Lovers' Day(情人节)

E: Feb 14, 2012.Valentine' Day

Valentine's Day(情人节), happy(快乐), ha-ha(哈哈), thanks(谢谢), together(一起) , happiness(幸福), gifts(礼物), lovers(礼物), chocolate(巧克力), festival(节日)

F: Mar 8, 2012. Women's Day

happy(快乐), festival(节日), Mother(妈妈), thanks(谢谢), women(女人), joyful(开心), March 8th(三八), girls(女生)

G: Apr 1, 2012. April Fools' Day

ha-ha(哈哈), happy(快乐), April Fools Day(愚人节)

H: Apr 3, 2012. Weibo opened comments to the users

comments(评论), Weibo(微博), finally(终于), the first comment(第一条评论)

I: May 13, 2012. Mothers' Day

Mother(妈妈), happy(快乐), Mothers' Day(母亲节), happiness(幸福), healthy(健康), I love you(我爱你), joyful(开心) , work hard(辛苦)

J: May 20, 2012. Day to speak out love

ha-ha(哈哈), happy(快乐), love(爱), I love you(我爱你), happiness(幸福), thanks(谢谢), fighting(加油), smile(笑), speak out of love(表白), fond of(喜欢)

K: Jun 1, 2012. Children's Day

happy(快乐), Children's Day(儿童节), care(留意), ha-ha(哈哈) , festival(节日), joyful(开心), gifts(礼物), kids(儿童), lovely(可爱), cute(萌)

L: Jun 8, 2012. Xin Liu's Birthday and the beginning of European Cup

birthday(生日), happy(快乐), Xin Liu(刘忻), thanks(谢谢); European Cup(欧洲杯), beginning(开幕)

M: Jun 23, 2012. Dragon Boat Festival and competitions of European Cup

happy(快乐), zongzi(粽子), ha-ha(哈哈) Dragon Boat Festival(端午节); Germany(德国), fighting(加油), Greece(希腊), European Cup(欧洲杯), field(赛场), best(最佳)

N: Aug 5, 2012. Dan Lin won the gold medal

fighting(加油), Dan Lin(林丹), China(中国), Super Dan(超级丹), Olympic Games(奥运会), Chong Wei Lee(李宗伟), Yang Sun(孙杨), congratulations(恭喜), Champaign(冠军), badminton(羽毛球)

O: Aug 23, 2012. Star Festival

Star Festival(七夕), happy(快乐), ha-ha(哈哈), Lovers' Day(情人节) , happiness(幸福), love(爱), smile(笑), together(一起), gifts(礼物)

P: Sep 30, 2012. Moon Cake Festival and the finals of the Voice of China

Mookcake Festival(中秋节) , mooncake(月饼) , go home(回家) , together(一起) , moon(月亮); Voice of China(好声音), Mochou Wu(吴莫愁) , fighting(加油), Bo Liang(梁博) , Jike Junyi(吉克隽逸)
