# Supplementary material for: Emoticon-Based Ambivalent Expression: A Hidden Indicator for Unusual Behaviors in Weibo
Source: PLoS One. 2016 Jan 22;11(1):e0147079. doi: 10.1371/journal.pone.0147079 (PMC4723056; doi:10.1371/journal.pone.0147079)
Supplement: S3 Table — (PDF) [file pone.0147079.s004.pdf]

Supporting Information Captions

S3 Table. Key terms for negative events.

A: Mar 31, 2012. Sina closed the comments in Weibo

comments(评论), reposts(转发), mean jokes(吐槽), Sina(新浪), rumor(谣言), close(关闭)

B: Apr 20, 2012. Volunteers rescued dogs in Kunming

dogs(狗狗), raining(下雨), rainstorm(暴雨), Guangzhou(广州), animals(动物), dog dealers(狗贩子), volunteers(志愿者), Kunming(昆明)

C: May 12, 2012. The 4th Anniversary of Wenchuan Earthquake

Four years(4 年), Wenchuan(汶川), victims(遇难者), rest in peace(安息), 5.12(512), earthquake(地震), survivals(生者), fellows(同胞), commemorate(纪念)

D: Jun 10, 2012. Group Stages in European Cup

Holland(荷兰), European Cup(欧洲杯), fighting(加油), Italy(意大利), Germany(德国队), Portugal(葡萄牙), Denmark(丹麦)

E: Jul 22, 2012. Torrential Rains in Beijing

Beijing(北京), rest in peace(安息), salute(致敬), heroes(英雄), rainstorm(暴雨), sacrifice(牺牲), death(死亡), local police station(派出所), policemen(警察)

F: Jul 27, 2012. Witness Insecurity

ending(大结局), Witness Insecurity(护花危情), Mr Xu(许 Sir), TVB(TVB), die(死了)

G: Aug 7, 2012. Xiang Liu Got Injured in the Olympic Games

Xiang Liu(刘翔), China(中国), London(伦敦), Olympic Games(奥运会), cry(哭), fail down(跌倒), referee(裁判), hero(英雄), fighting(加油)

H: Sep 30, 2012. The final of Voice of China

Voice of China(中国好声音), Mochou Wu(吴莫愁), ads(广告), Zhiwen Jin(金志文), shady deal(黑幕), Bo Liang(梁博), Sherry Chang Huei-mei(张惠妹)
